# Vestibular evoked myogenic potentials: an overview

**DOI:** 10.1016/S1808-8694(15)30666-2

**Published:** 2015-10-19

**Authors:** Renato Cal, Fayez Bahmad

**Affiliations:** 1 Otolaryngologist. Researcher at the Department of Otology - Massachusetts Eye and Ear Infirmary, Harvard Medical School, Boston, MA, EUA; 2 Otolaryngologist Researcher at the Department of Otology - Massachusetts Eye and Ear Infirmary, Harvard Medical School, Boston, MA, EUA. Assistant Physician - Department of Otorhinolaryngology and Head and Neck Surgery - Hospital Universitário da Universidade de Brasília. Graduate Student (PhD0 in Medical Sciences - Universidade de Brasília (UnB), Brasília, DF, Brazil. Harvard Medical School, Boston, MA, EUA. Departamento de Otorrinolaringologia e Cirurgia de Cabeça e Pescoço do Hospital Universitário de Brasília, Brasília, DF, Brazil

**Keywords:** endolymphatic hydrops, evoked potentials, vestibular

## Abstract

The vestibular evoked myogenic potential (VEMP) test is a relatively new diagnostic tool that is in the process of being investigated in patients with specific vestibular disorders. Briefly, the VEMP is a biphasic response elicited by loud clicks or tone bursts recorded from the tonically contracted sternocleidomastoid muscle, being the only resource available to assess the function of the saccule and the lower portion of the vestibular nerve.

**Aim:**

In this review, we shall highlight the history, methods, current VEMP status, and discuss its specific application in the diagnosis of the Ménière's Syndrome.

## INTRODUCTION

Vestibular evoked myogenic potentials (VEMP) are inhibitory electrical potentials generated after a sound stimulus (clicks or pure tones), originated in the saccule and conducted by the lower portion of the vestibular nerve all the way to the central nervous system (CNS), generating inhibitory electrical responses picked up by electrodes placed on the sternocleidomastoid muscle (SCM)^1^,^2^. In English, these potentials are known by the acronym VEMP, meaning Vestibular Evoked Myogenic Potentials. The reason why we have had a growing interest on this topic in recent years is because of the physiological origin (saccule and lower division of the vestibular nerve) of these potentials and their possible clinical applications.

Under the evolutionary viewpoint, the cochlea was the last portion to develop within the membranous labyrinth^3^, ^4^, ^5^, which suggests that in some species of fish and even in some mammals, the saccule also has an auditory function, causing some authors to speculate about the existence of some sensorial cells to sound stimulus in the saccule of human beings^4^,^6^,^7^. Still considering this evolutionary characteristic, it is believed that when we are submitted to a high intensity sound stimulus, these remaining saccule cells are stimulated, triggering an inhibitory reflex to the ipsilateral SCM muscle, causing its relaxation with consequent contraction of the contralateral muscle.

It is exactly this unilateral inhibitory reflex which we capture with electrodes positioned on the SCM muscle that we know as VEMP. These are exactly the remaining hair cells in the saccule that would originate VEMP, and this stimulus is taken to the CNS through the lower portion of the vestibular nerve (IVN)^2^,^7^, ^8^, ^9^, ^10^. This makes VEMP a new method that neurotologists have at hand to diagnose and investigate the vestibular system, because until then there was no exclusive way to assess saccule and IVN function.

## OBJECTIVE

The goal of the present review is to highlight the history, modus operandi, current status of the research involving VEMPs, and also to discuss its specific applications in the diagnosis of Ménière's syndrome, vestibular schwannoma, superior semicircular canal dehiscence syndrome, perilympathic fistula, vestibular neuritis and other vestibular lesions, in an attempt to help otolaryngologists and neurologists work with this new diagnostic tool.

## MÉTHOD

Based on a broad literature review - the authors used the MEDLINE database (www.pubmed.com), and on the clinical experience acquired in a reference tertiary hospital for neurotology disorders in the USA, they carried out an updated analysis on VEMPs.

## RESULTS

### Background

The first sound evoked vestibular responses were described by Von Békésy in 1935. He used high intensity sound stimuli (about 134 dB) in order to generate head movements towards the sound stimulus7. The explanation for this phenomenon was the proximity between the stapes footplate and the saccule macula hair cells, which would activate the afferent neurons. This proximity between the stapes footplate and the saccule macula has been broadly described in the international literature, by means of macro and microscopic anatomical studies of the temporal bone^11^.

In 1964 we had the first reports on short-latency myogenic electrical evoked potentials^12^,^13^; however, it was only 7 years later that Towsend et al. noticed that the true origin of these potentials was the saccule^14^. The authors proved it when they found VEMPs present in deaf patients; however, they were absent in patients submitted to vestibular neurectomy. Later, these same authors discovered that VEMP responses were present in patients who had been submitted to ablation of the semicircular canals by streptomycin and in patients, who had benign paroxysmal postural vertigo (BPPV), while these same potentials were absent in patients with Ménière's syndrome^14^.

In order to reinforce the physiological basis of VEMPs, McCue & Guinan identified some fibers from the inferior division of the vestibular nerve in cats, which responded electrically to sound stimuli above 80 dB SPL (sound pressure level), which increased their electrical activity as the stimulus intensified^15^,^16^, confirming the hypothesis that these muscle inhibitory electrical potentials were originated in the saccule and, consequently, had their afferent pathways in the IVN.

**Figure 1:**
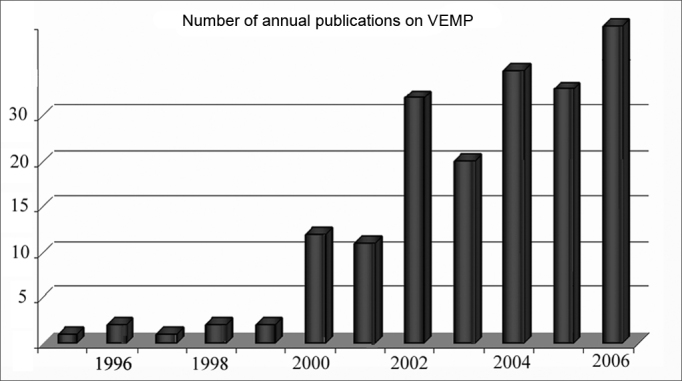
Annual publications on the Vestibular Evoked Myogenic Potential (VEMP) since 1995. Notice that as of the year 2000, there was a quick increase in the number of publications dealing with VEMP use.

Source: www.pubmed.com.

Since then, the interest for this topic has been on the rise, with a significant increase in the number of publications in recent years about VEMPs^1^,^7^,^17^, ^18^, ^19^, ^20^, ^21^, ^22^, ^23^. It was only in 2005 that more than 30 papers were published in English2, and in 2006 this number went up to 33 (source www.pubmed.com). It is believed that in 2007, this number will be even greater (Figure 1).

### VEMP test performance

**Figure 2a:**
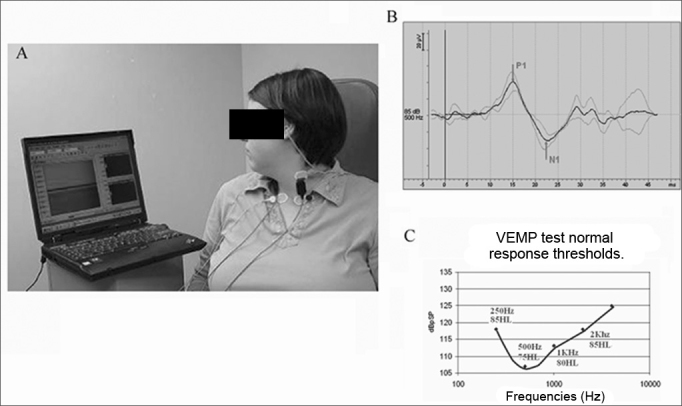
VEMP test on the left side with earbud phones and electrodes placed on the SCM. The patients are placed on a comfortable chair and are instructed to turn their heads to the direction contrary to that of the sound stimulus, so as to have SCM muscle contraction to the ipsilateral side of the sound stimulus. **Figure 2b**. VEMP typical wave in a normal patient. Showing the first positive wave (P1) around 13ms and the second negative wave (N1) around 23ms. **Figure 2c**. Typical VEMP curve in a normal patient, showing the response threshold of the main frequencies tested.

These potentials are generated after high intensity sound stimuli (clicks or pure tones), of 0.1 msec of duration, at about 140 to 145 dB SPL (about 90 dB hearing level), sounded in an ear phone (monaural or bilateral). The patients are put in a chair, seated and instructed to turn their heads to the opposite side of the sound stimulus, in order to contract the contralateral SCM muscle. VEMPs are read by electrodes placed on the patient's SCM (ipsilateral to the sound stimulus), the positive electrode is placed on the upper third of the muscle, while the negative electrode is placed on the muscle tendon, just above the clavicle (Figure 2A).

This electrode placement can happen in an inverse way, causing response wave inversion. The ground electrode is usually positioned on the patient's vertex. Although the SCM muscle is the most frequently site used to obtain vestibular responses, some authors attained these responses from other muscles such as the trapezius and muscles in the legs and arms^3^,^24^.

It is worth to bear in mind that one needs a normal middle ear in order to be able to record VEMPs, since small air-bone gaps of 8.75 dB are enough to dampen the responses^25^. The electrical responses from these potentials are made up of two biphasic waves, the first is positive, with a latency around 13ms, known as p13; followed by another wave, this time negative, with a latency around 23ms, known as n23 (Figure 2B).

These responses are present in most of the normal individuals studied^5^, differently from a second biphasic complex known as n34-p44, which according to Colebatch would be absent in 40% of the normal individuals studied^26^, while Robertson described that this second complex would be present in 68% of normal individuals27. Because of the lack of reproducibility of this second complex (n34-p44), the vast majority of these studies with VEMP only considers the first biphasic complex (p13-n23)^1^,^2^,^7^, ^8^, ^9^, ^10^,^17^,^26^,^28^.

One of the most controversial points regarding VEMPs is the relationship observed between the electrical response amplitudes and the level of contraction of the muscles tested (usually the SCM). This relationship was noticed by Colebatch et al., in 1994 and confirmed by many other studies^1^,^2^,^5^,^7^, ^8^, ^9^,^26^. This relationship is relevant because we would need standardized muscle contractions among the patients, and if it does not happen, it becomes very hard to be able to compare the test of an elderly patient with that from a young athlete, who very likely has a much higher level of muscle contraction. Thus, many studies are currently used to monitor SCM contraction, such as electromyography and biofeedback^5^,^7^,^23^.

Another controversial point regarding VEMPs is that the sound stimulus is generated through clicks or tone bursts. Many authors study this topic^9^,^10^,^29^. In 2003, Cheng et al. did a study in which they compared the influence of clicks and tone bursts in the generation of vestibular responses. They concluded that VEMPs generated by clicks have a greater response index in normal individuals, greater amplitude of waves p13-n23 and lower latency^9^,^29^. These data show that when the physician is reading a VEMP test, he/she must know which was the sound stimulus used, since it impacts directly on wave amplitude and latency. Nonetheless, in 2004 Rauch et al. showed that within the range of frequencies tested to generate VEMP responses, 500 Hz was the most sensitive. Moreover, he introduced a new way of analyzing VEMP results, by studying not only amplitude and latency, but also the response threshold in four different frequencies (250, 500, 750 and 1000Hz)^30^. (Figure 2C)

So far, there are no specific devices to be used in VEMP testing, and most of the Centers use the ABR equipment (audiometric analysis of the auditory nerve and brainstem) to record these responses. Some authors also use other means to trigger these potentials in patients with conductive hearing loss, they use a bone stimulus by gently tapping the skull of the patients with a small hammer^31^. They attained responses in 10ms (positive wave) and 17ms (negative wave) in patients in whom the sound stimulus was incapable of generating such responses because of their conductive hearing loss.

### Clinical applicability

Differently from what happens in the auditory system, the vestibular system is much more complex and much less known and studied, that is why the interest on VEMPs has grown in recent years, because these potentials reflect the function of the otolithic organs (saccule) and the IVN, something that so far was not possible to be evaluated by the numerous other vestibular tests, such as vector-electronystagmography, the rotating chair test and the platform test^2^.

The caloric test which, without any doubt is the most used test to check vestibular function, is limited to the workings of the lateral semicircular canal and the upper portion of the vestibular nerve in each side separately, while the platform test does not assess each one of the labyrinths separately, nor one of the systems in charge of body balance (vision, labyrinth and proprioception), separately.

Many publications have shown VEMPs used as a means to diagnose or even to help diagnose the most diverse neurotological diseases, such as Ménière's disease, superior semicircular canal dehiscence, vestibular neuronitis, vestibular schwannomas, control after intratympanic administration of gentamicin and even perilymphatic fistulas^1^,^2^,^5^,^7^,^19^,^28^,^30^,^32^,^33^. The role of VEMPs in each one of these situations is described as follows:

### Vestibular Neuronitis

It is one of the most frequent causes of vertigo and it is normally diagnosed based on the patient's clinical history, associated with a caloric test that shows a unilateral functional deficit. 34 According to previously held studies, we know that both branches of the vestibular nerve (inferior and superior) can be affected by this disease; however, based on studies by Goebel et al. in 2001, we can have some explanations about the possible reason why the upper portion is much more frequently affected than its inferior counterpart^35^.

It is exactly based on this differentiation between the upper and lower branches that VEMPs can be utilized. In 1995, Halmagyi et al. studied 22 patients with diagnosis of vestibular neuronitis and did not get caloric responses in the affected sides of these patients. Curiously, he observed that when submitted to VEMP testing, the responses were normal in 6 patients, reduced in 5 and absent in 1133. This proves that some patients have both portions of the vestibular nerve involved, while others have lesions in its upper portion only. Such fact is very important regarding the prognosis of these patients, since the patients with absent or altered VEMPs will hardly develop BPPV in the posterior semicircular canal, since the nerve that innervates this canal is damaged (IVN). Now, those patients with manifestations of vestibular neuronitis and normal VEMPs show that upper division of the nerve was involved, it is possible that patient will develop posterior canal BPPV in the future^36^.

### Superior Semicircular Canal Dehiscence

In 2001, researchers from the Johns Hopkins University in the United States, headed by Professor Lloyd B. Minor, described a vestibular entity not previously reported in the literature, called superior semicircular canal dehiscence^37^. This syndrome is characterized by vertigo and nystagmus triggered by sound and/or pressure stimuli, and its pathophysiology is based on the “Third Window” theory. The existence of this third window in the internal ear (besides the round and oval windows) would cause an impedance reduction in the inner ear, allowing a better membranous labyrinth fluid movement and consequently a greater deflection of the vestibular sensors to sound and pressure stimuli^37^, ^38^, ^39^, ^40^.

Based on this physiopathological explanation, VEMPs have become rather important, since the existence of this third vibratory window would cause a greater movement of the stapes on the oval window, and consequently a greater stimulation on the saccule macula to the sound stimuli employed. This would bring down VEMP thresholds in almost all the frequencies tested, and such data has been confirmed by numerous studies, such as the one carried out by Brantberg et al. in 1999, which tested VEMPs in 3 patients with superior semicircular canal dehiscence, noticing a threshold reduction in the affected side of all patients, especially within the range between 500 Hz to 1000 Hz^41^.

A similar study was carried out by this same group in 2001, when 8 patients with superior semicircular canal dehiscence syndrome were submitted to VEMP testing and all had threshold reduction in the affected side; 4 of the 8 patients tested had normal audiometry and 6 of the 8 had normal responses in the caloric test^42^. These studies reinforce the importance of VEMPs in the diagnosis of this syndrome that often times has a varied clinical manifestation, normal audiometric findings or findings that mimic otosclerosis (conductive hearing loss) and that can only be confirmed by means of a high resolution CT-scan^37^, ^38^, ^39^,^42^.

### Vestibular Schwannoma

This benign tumor which affects the vestibular pathways is always a diagnostic hypothesis considered in the most varied clinical cases. Currently, the most efficient method to diagnose it, and also the most expensive one is Magnetic Resonance Imaging (MRI). Nonetheless, because of its high cost and the impossibility of performing the test in all suspected patients, the ABR also plays an important role in the diagnosis of vestibular schwannomas^5^.

Knowing that VEMP neural pathways involve the inferior portion of the vestibular nerve, this diagnostic method can also be used to help in the diagnosis of vestibular schwannomas. Some studies in the literature already show this important contribution of VEMPs, such as the one developed by Murofushi et al. in 1998, who observed 80% of altered VEMPs in 17 patients with established diagnosis of vestibular schwannoma^43^. In 2001, Takeichi et al. performed a similar study in which he observed altered VEMPs in 13 of 18 patients diagnosed with vestibular schwannoma confirmed by MRI^44^.

In a general way, VEMPs can contribute to the diagnosis of vestibular tumors, but must not be used as the sole diagnostic method, because it only assesses the function of the inferior vestibular nerve. Nonetheless, when performed together with MRI, the ABR, audiometry and caloric test, they may help in the exact location of the tumor in the vestibular pathways.

### Ménière's Syndrome (MS)

Among all the clinical applications of VEMPs, this can be the one with the greatest clinical relevance. Besides prior studies in temporal bones, the relationship between the endolymphatic hydrops and MS is well established^45^, ^46^, ^47^, ^48^, ^49^, ^50^. Schuknecht et al. showed that the most frequently involved sites by endolymphatic hydrops are the cochlea, followed by the saccule and the utriculus, respectively^11^,^46^,^47^,^49^,^51^. Since the saccule is considered the site of origin of VEMPs, it is expected to find VEMPs altered even in early stages of the Ménière's Syndrome.

In 2004, Rauch et al. published a study in which they showed VEMPs present in about 94% of the patients with MS in the affected side and the frequency thresholds between 250 Hz and 2,000 Hz were increased^30^. Nonetheless, one of the most interesting pieces of data of this study was the fact that about 27% of the asymptomatic ears from these patients with unilateral MS had alterations in this test. When we compared this result to data from temporal bone studies that found about 38% of endolymphatic hydrops in asymptomatic ears of patients with MS1 and epidemiological studies that reported bilateral involvement of MS in 30% to 35% of the patients, we concluded that VEMPs may be a diagnostic method for endolymphatic hydrops in initial stages, and it can serve as a prognostic factor for bilateral involvement in MS.

Many other studies were also published in the international literature recently about VEMPs in patients with MS. In 2001, Murofushi et al. reported that 51% of the patients with MS had no response to the VEMP test^52^, while Waele et al., doing the same study, observed an index of 54% of response absence in the VEMP test in patients with MS^53^. Other authors, performed studies comparing the relationship between the glycerol test and VEMPs, concluding that VEMP results after administering glycerol were altered, and VEMP testing is the only useful method to diagnose endolymphatic hydrops^54^,^55^.

### Perilymphatic Fissure

In 2006, Modugno et al. from the University of Bologna, Italy, published a study in which VEMP testing was used for the diagnosis of endolymphatic fistula cases^56^. They reported four cases in which VEMP response thresholds were reduced with stimuli in the frequency of 500Hz. The possible explanation for this threshold lowering is based on the same theory that explains this phenomenon in cases of superior semicircular canal dehiscence, a third vibratory window in the internal ear would reduce the impedance, consequently causing a reduction in these thresholds. One important criticism to this study published in 2006 is the small number of patients tested and the absence of a longer follow up.

### Monitoring after treatment with intratympanic gentamicin

In recent years, the Ménière's disease treatment in cases resistant to clinical treatment started to be carried out through the administration of intratympanic gentamicin, in an attempt to reduce vertigo symptoms in these patients^57^. After this therapy, many professionals performed an electronystagmography with caloric test in an attempt to confirm if the gentamicin dose employed was enough to cause damage to the vestibular cells and it is exactly with this aim that VEMPs can also be used.

In 2002, De Waele et al., showed that 92% of the patients submitted to intratympanic injection of gentamicin had no responses in VEMP testing in one month and they kept like that for even one year after treatment^58^.

## FINAL REMARKS

VEMP testing is a new complementary test, which may contribute, together with other neurologic tests, for the diagnosis of many and diverse vestibular disorders, among them we stress: Ménière's Syndrome and Superior Semicircular Canal Dehiscence.

The test is currently used in tertiary reference centers that deal with neurotological disorders. Nonetheless, the authors stress that since this is a new diagnostic method, the use of VEMPs is still not totally adapted to clinical use, since most of the facilities still don't have their own equipment (using the same ABR equipment), trained professionals for their proper realization and interpretation, and lack international standardization to analyze results.

Another extremely important point to be highlighted is the need for standardization in relation to monitoring SCM muscle contraction level, before VEMPs are employed in clinical use, since VEMP responses are broadly dependent on the level of activities of this muscle.

At any rate, VEMP testing seems to be a promising complementary test, especially for providing information on the function of the saccule and inferior portion of the vestibular nerve.
